# First Report on a Solvent-Free Preparation of Polymer Inclusion Membranes with an Ionic Liquid

**DOI:** 10.3390/molecules24101845

**Published:** 2019-05-14

**Authors:** Ruben Vera, Enriqueta Anticó, José Ignacio Eguiazábal, Nora Aranburu, Clàudia Fontàs

**Affiliations:** 1 Department of Chemistry, University of Girona, C/Maria Aurèlia Capmany 69, 17003 Girona, Spain; ruben.vera@udg.edu (R.V.); enriqueta.antico@udg.edu (E.A.); 2 Departamento de Ciencia y Tecnología de Polímeros, Instituto de Materiales Poliméricos “POLYMAT”, Universidad del País Vasco, 20080 San Sebastián, Spain; nora.aramburu@ehu.eus

**Keywords:** polymer inclusion membrane, thermal-compression technique, thermoplastic polymers, Aliquat 336

## Abstract

A novel and environmentally-friendly procedure for the preparation of polymer inclusion membranes (PIMs) containing an ionic liquid is presented for the first time. Traditionally, PIMs are prepared by a solvent casting method with the use of harmful organic solvents. Here we report a new solvent-free procedure based on a thermal-compression technique which involve the melting of the components of the PIM and the application of a high pressure to the melted specimen to form a flat-sheet film. In our study, we have tested different polymers, such as two cellulose derivatives as well as two thermoplastic polymers, polyurethane (TPU) and poli ε-caprolactone (PCL). The ionic liquid (IL) trioctylmethylammonium chloride (Aliquat 336) has been used to produce PIMs with a fixed composition of 70% polymer–30% IL (*w*/*w*). Both TPU and PCL polymers provide successful membranes, which have been thoroughly characterized. PIMs based on the polymer PCL showed a high stability. To test whether the properties of the IL were affected by the preparation conditions, the extraction ability of Aliquat 336 was investigated for both PCL and TPU membranes in terms of Cr(VI) extraction. Satisfactory values (90% extraction) were obtained for both membranes tested, showing this novel procedure as a green alternative for the preparation of PIMs with ILs.

## 1. Introduction

Membrane-based processes have attracted a great attention during the last decades as a valuable alternative for many separation applications [1,2]. Among the different types of membranes, polymeric membranes incorporating ionic liquids (ILs) are under the spotlight due to the satisfactory properties of these compounds. ILs are molten salts, liquid at room temperature, formed by an organic cation (e.g., dialkylimidazolium, tetralkylammonium, trycaprylmethylammonium, among others) and an organic or inorganic anion. ILs exhibit certain remarkable features such as negligible vapour pressure, high ion conductivity, low volatility, non-flammability, liquid range of up to at least 300 °C, high potential for recycling, high solvating capacity and high viscosity, etc. [3,4]. However, the use of ILs presents some limitations due to, for example, their high price for synthesis and high energy consumption for recycling, which can be overcome by including them into polymeric membranes, where a less amount of IL is required [4].

Polymer inclusion membranes (PIMs) are a type of polymeric membranes where the extractant is immobilized between the entangled chains of the membrane’s base polymer. PIMs have successfully been used in different separation systems, including analytical applications [5], transport of organic compounds [6,7], as well as metallic species [8,9].

The IL trioctylmethylammonium chloride (Aliquat 336) is widely used to produce membranes due to its versatility and affordability, and has successfully been employed as a cation source of a new family of hydrophobic ILs [10]. Its ability to extract anionic species has been exploited in several studies concerning either inorganic [11,12,13,14,15] and organic compounds [16,17].

PIMs are conventionally prepared by the solvent casting method, where the polymer and the extractant are properly dissolved in an appropriate volatile solvent, which afterwards, is allowed to evaporate to form a thin stable polymeric film. Another approach is the phase inversion technique, which involves several steps, such as the dissolution of the polymer and extractant in a solvent, the spreading of this solution onto a substrate, and the solvent removal by immersing the nascent membrane in water [11]. Even though the simplicity of these procedures, they present the important drawback of requiring the use of harmful organic solvents. This is the case when the polymers cellulose triacetate (CTA) or polyvinyl chloride (PVC) are used. In the first case, CTA needs solvents such as chloroform or dichloromethane (10 mL for 0.1 g of polymer) [8,18,19], whereas PVC is dissolved using tetrahydrofuran (5 mL for 0.1 g of polymer) [20,21]. These two polymers, and more recently poly(vinylidine fluoride-cohexafluoropropylene) (PVDF-HFP), which needs to be dissolved in THF, are the most employed polymers to produce PIMs.

Recent research on the preparation of polymeric membranes is focused on the use of polymers that need less harmful reagents such as the case of Pebax 1657, which can be dissolved in a mixture of ethanol and water at a high temperature [22] or in 1-butanol [23]. Membranes prepared with this polymer and the IL based on the cation 1-butyl-3-methyl imidazolium ([Bmim+]) combined with different anions are obtained by the solvent casting method, and have shown a good efficiency in terms of CO_2_ separation. Additionally, a review on different non-toxic solvents used for the preparation of polymeric membranes has recently been presented by Figoli et al. [24]. This review highlights the possibility to use less toxic organic solvents such as methyl or ethyl lactate, triethylphosphate, dimethyl sulfoxide or γ-butyrolactone as safer and attractive alternatives to dissolve polymers, such as cellulose acetate (CA) or poly(vinylidene fluoride) (PVDF), to produce membranes for ultrafiltration, microfiltration, or reverse osmosis processes.

As a further step to achieve greener separation processes based on membranes, we have explored, in this study, a new methodology avoiding the use of hazardous chemicals to prepare PIMs incorporating an IL. Moreover, we have also investigated, for the first time, the use of eco-friendly polymers for this purpose. This is the case of the thermoplastic polymers polyurethane (TPU) and poli ε-caprolactone (PCL). TPUs are linear block copolymers containing hard and soft segments. According to the manufacturer, TPU used in this work is a linear, aromatic bio-polyurethane based on specialty polyol from renewable sources (67% renewable material) with an extremely high crystallization rate and a very high thermoplasticity level. TPU polymers show a wide range of properties, making them suitable for different applications including automotive, electronics, sports goods, footwear and medical applications [25]. The polymer PCL is a biodegradable high molecular weight linear polyester derived from caprolactone monomer with a great potential to be used as an implantable biomaterial in the biomedical field [26]. It must be highlighted that the biodegradability of this polymer will reduce its overall environmental impact, such as the formation of microplastics, which is of a great concern nowadays. To the best of our knowledge, this is the first study on the preparation of PIMs incorporating ILs by a thermal-compression procedure. The introduction should briefly place the study in a broad context and highlight why it is important.

## 2. Results and Discussion

### 2.1. Preparation of Polymeric Membranes

As stated in the introduction, PVC and CTA are widely used in the preparation of PIMs. However, both polymers present high melting temperatures and are not appropriate for the thermal-compression method due to the high temperatures required [27] that would lead to the degradation of the IL. Given this, instead of CTA, two cellulose acetate derivatives, CAP and CAB, that have also been used to produce PIMs by the solvent casting method [28,29] were selected due to their more adequate melting points. Moreover, membranes based on TPU and PCL were also tested.

The characteristics of the membranes prepared by the thermal-compression technique are collected in Table 1. Due to the high processing temperature needed to prepare membranes using CAP, the resulting film was not mechanically stable. Additionally, the brownish colour can be related to the IL degradation. Hence, this polymer was discarded for further experiments. When CAB was employed as the base polymer, the degradation of the IL was not observed, but the resulting membranes presented an oily surface, a lack of flexibility and a brittle behaviour. The presence of oil droplets on the surface is an indicator that the IL is not completely entrapped into the polymeric matrix as a result of a low compatibility between the polymer and the IL [30]. Non-oily and flexible membranes were obtained with both polymers TPU and PCL. Taking into account that satisfactory membranes are expected to incorporate the IL without its degradation, as well as to show a mechanical strength to facilitate the manipulation of the membrane, TPU and PCL were selected as polymers to produce membranes in further experiments.

### 2.2. Membrane Characterization

Elemental analysis of TPU and PCL membranes was performed and results are presented in Table 2. A good agreement was found between the theoretical expected values (calculated considering the mass of each component of the membrane) and the experimental ones for the atomic content of C, H and N. It is important to point out that N is only present in the IL in the case of membranes prepared with PCL whereas a 4% of this atom is also present in the formulation of TPU polymer. The fact that N (%) found in the membranes corresponds to the expected values confirms that the IL has been satisfactorily incorporated in the membrane.

**Figure 1:**
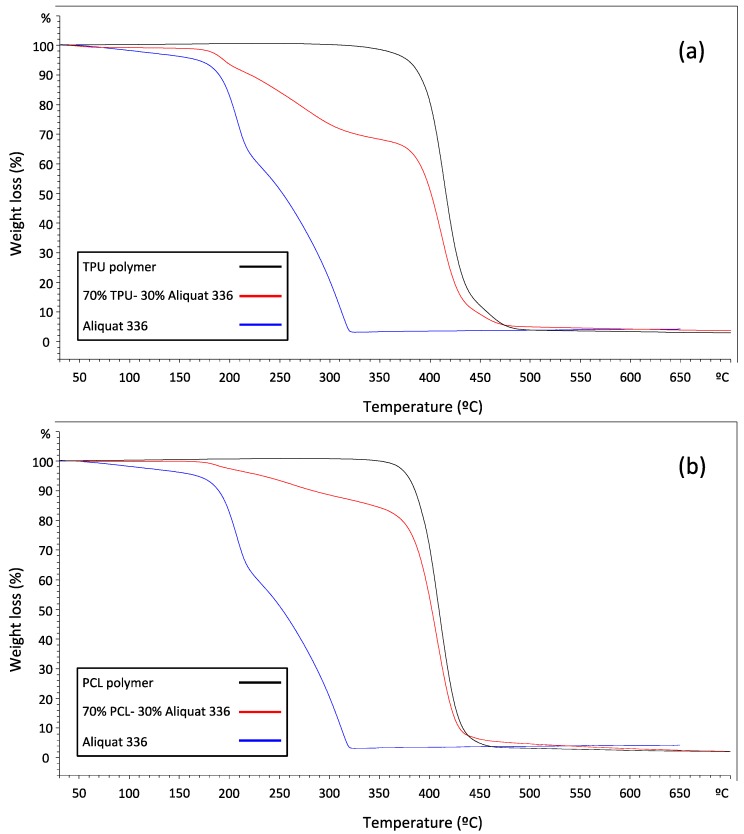
TGA curves for 70% TPU–30% Aliquat 336 (**a**) and 70% PCL–30% Aliquat 336 (**b**).

PIMs were also investigated by TGA analysis. TGA curves are presented in Figure 1 including both obtained for the membrane and the corresponding to the pure components. The TGA curve of the membrane 70% TPU–30% Aliquat 336 clearly shows three parts: a first decrease of the total weight loss (around 4%) at temperatures up to around 100 °C, which corresponds to the loss of volatile materials and water absorbed by the membrane. Secondly, the loss starting at a temperature of 180–200 °C that can be attributed to Aliquat 336 thermal decomposition, which is also observed in the Aliquat 336 TGA curve (blue line) and represents a weight loss of 30%. Finally, the third decrease in the membrane TGA curves at temperatures over 320–330 °C, which is associated to the thermal degradation of the polymer TPU. These results are in concordance with other studies where PIMs containing Aliquat 336 were prepared by solvent casting [31]. The TGA curve of the membrane 70% PCL–30% Aliquat 336 also presents three parts. However, the weight loss corresponding to the IL represents a 20% of the total value instead of the expected 30%. This result was confirmed by analysing different new segments of the membrane. This fact seems to indicate that Aliquat 336 degradation is shifted to further temperature and cannot be isolated from the polymer decomposition, likely due to some interactions in between the polymer and the IL.

**Figure 2:**
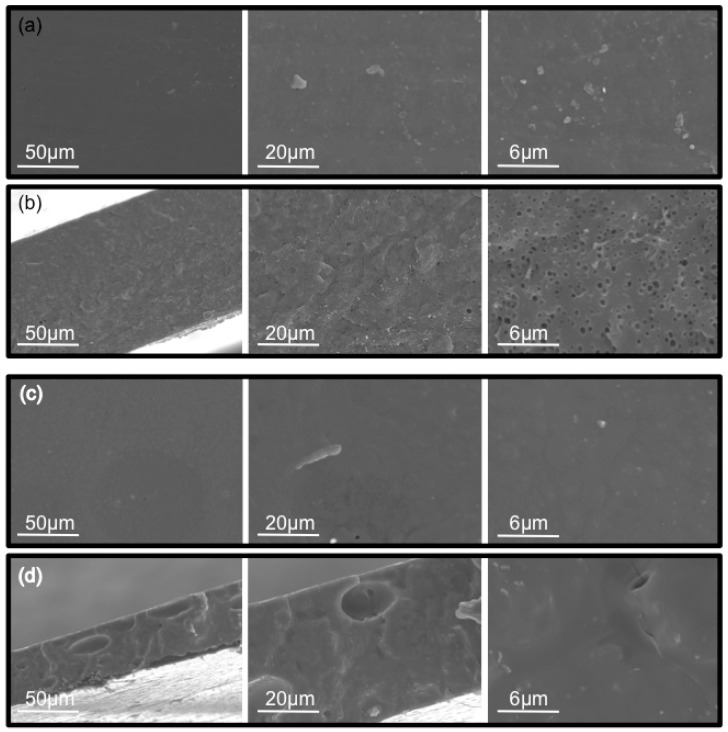
SEM images of PIMs prepared by thermal-compression technique. 70% TPU–30% Aliquat 336: surface (**a**), cross-section (**b**); 70% PCL–30% Aliquat 336: surface (**c**), and cross-section (**d**).

Morphological characterization of PIMs was performed by SEM analysis. From the surface images of both TPU (Figure 2a) and PCL (Figure 2c) membranes, it can be observed that all membranes present a uniform surface, dense and with no apparent porosity. However, cross-section images of the membranes revealed a non-homogeneous structure. In the case of TPU membranes (Figure 2b), it can be observed a porous structure with pores size < 600 nm, and membrane thickness of 125 μm, whereas a dense structure without pores but presenting some cavities (ranging from 20 up to 50 μm) are found in PCL membranes (Figure 2d) and membrane thickness of 50 μm. It is known that this later polymer forms porous membranes, also distinguishable on surface images [26,32]. The fact that pores are not present on the surface of membranes containing an IL and prepared by thermo-compression seems to indicate a great entanglement of Aliquat 336 within the polymeric matrix, producing a dense uniform structure. However, the presence of cavities in the body of the membranes can be attributed to tiny air bubbles, which are entrapped into the highly viscous mixture during the mixing step by an extruder.

**Figure 3:**
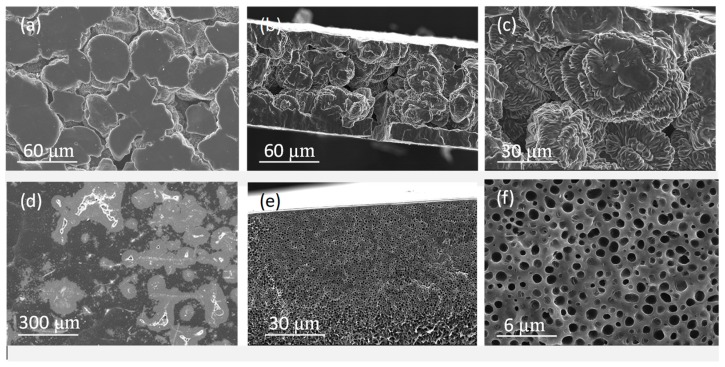
SEM images of PIMs prepared by the solvent casting method. 70% TPU–30% Aliquat 336: surface (**a**), cross-section (**b**,**c**); 70% PCL–30% Aliquat 336: surface (**d**), and cross-section (**e**,**f**).

Moreover, for comparison purposes, PIMs of the same composition and using the polymers TPU and PLC were also prepared by the solvent casting method. Figure 3 shows the morphology of the resulting membranes. As it can be seen, the resulting membranes are very different from those prepared by the thermal-compression technique. In the case of TPU, the membranes were not homogeneous, presenting some aggregations of the material which can be appreciated both on the surface and in the cross-section of the membrane. When PCL was used as the polymer, a homogeneous and highly porous film was obtained, with pores size ranging from 0.6–0.9 µm. This extremely porous structure restrains the use of these membranes for transport experiments, since PIMs are expected to not allow the flow of water through them, as it happened for this membrane.

### 2.3. Stability Studies

It is well-known that an important drawback of PIMs incorporating slightly soluble ILs, such it is the case of Aliquat 336, is their lack of stability due to the solubilisation of these compounds into the aqueous solutions. This fact is strongly related with the loss of efficiency of the membrane. Hence, we investigated the stability of the membranes when contacted in ultrapure water. Membrane’s mass loss, which is assumed to correspond to the loss of the IL, was evaluated, and it was found that PCL-based membranes prepared by the thermal-compression technique appeared to be the most stable with only a 7.1% ± 0.7% in mass variation, while this value increased up to 35% ± 2% for PIMs prepared by the solvent casting method. For TPU, the mass loss was 23% ± 2% for membranes prepared by thermal-compression and 34% ± 1% when using this polymer for the solvent casting method. The different behaviour of the IL entrapped into the PCL matrix when the thermal-compression technique is used evidences certain interactions between these two components, as it was previously observed in TGA analysis. It is important to highlight that, for the first time, we report the production of PIMs based on a biodegradable polymer using a solvent-free preparation procedure and presenting an outstanding high stability.

### 2.4. Testing Membranes Efficiency: Cr(VI) Extraction

In order to assess whether the high processing temperatures required for the PIM preparation by the thermal-compression technique affected the performance of the IL, we tested the extraction abilities of Aliquat 336 towards Cr(VI) anions since it is well-known that Aliquat 336 interacts very effectively with this anion according to the formation of an ion-pair, as shown in Equation (1) [13,33]:

HCrO_4_^−^ + [Alq^+^Cl^−^] ↔ [(Alq^+^) HCrO_4_^−^] + Cl^−^ (1)

where Alq^+^Cl^−^ represents Aliquat 336.

**Figure 4:**
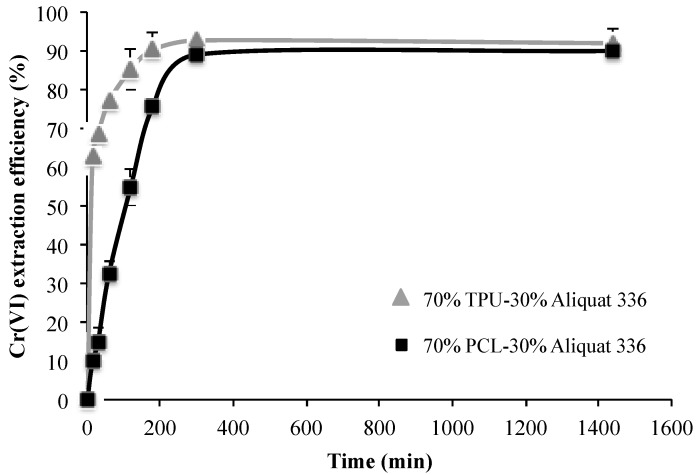
Transient Cr(VI) extraction at pH 4 using two different PIM compositions (n = 2).

The extraction of Cr(VI) was evaluated for both PIMs over time, and results are presented in Figure 4. It is noticeable the fast and efficient extraction exhibited by TPU membranes, with an extraction value of 60% in only 15 min, being 10% when the polymer was PCL. However, after 5 h of contact time a quantitative extraction was achieved in both cases. This different behaviour at a short time period supports the fact that the IL is differently entangled depending on the polymer.

## 3. Materials and Methods

### 3.1. Reagents and Solutions

The polymers CTA, cellulose acetate propionate (CAP), cellulose acetate butyrate (CAB) were purchased from Sigma-Aldrich (Steinheim, Germany). Polyurethane Pearlbond ECO D-590 (TPU) was obtained from Lubrizol (Barcelona, Spain) and poli ε-caprolactone (PCL) from Perstorp (Malmö, Sweden). Melting points, chemical structures and processing temperatures of the different polymers are depicted in Table 3.

Aliquat 336 (Sigma-Aldrich, Steinheim, Germany) was used as the IL to be incorporated in all polymeric membranes.

A stock solution (1000 mg L^−1^) of Cr(VI) was obtained from the solid K_2_Cr_2_O_7_ (Panreac, Barcelona, Spain) and was used to prepare working solutions containing 10 mg L^−1^ Cr(VI) at pH 4 (adjusted by using HCl (Panreac, Barcelona, Spain)). Calibration standards of Cr were prepared using Reagecon Chromium ICP standard solution. All solutions were prepared using analytical reagent grade chemicals and ultrapure water from a Milli-Q Plus water purification system (Millipore Ibérica S.A., Barcelona, Spain).

### 3.2. PIM Preparation

Membrane composition was fixed at 70% polymer and 30% IL (% in mass). This amount of IL has been demonstrated to provide satisfactory results in other studies [20]. Prior processing, the polymers TPU and PCL were dried in an oven at 60 °C overnight, following the recommendations of the manufacturer, whereas CAP and CAB were used as received. For the thermal-compression technique preparation, in all cases, 2.8 g of polymer and 1.2 g Aliquat 336 were mixed by means of a mini-extruder (DSM MICRO 5 cc) under the following experimental conditions: spindle and nozzle temperature were set at the corresponding processing temperature for each polymer (see Table 3) and spin speed of the spindle at 100 rpm. The extruded mixture was then placed between two metallic plates (hot-press (Collin P200E)), which were warmed up to the corresponding processing temperature (see also Table 3) for 2 min before placing the blend on the two metallic plates. After this period, the extruded mixture was placed in the hot press and no pressure was applied for 3 min to allow the total melting of the product. An increasingly pressure of 50 bars was then applied every 15 s up to 200 bars, and maintained for 2 min. Finally, the system was cooled down to room temperature and a flat membrane was obtained.

For comparison purposes, membranes made of PLC and TPU were also prepared by the solvent casting method [19,34].

### 3.3. Membrane Characterization

Elemental analysis of the PIMs was performed using a Perkin Elmer EA2400 instrument (mineralization temperature: 925–930 °C). Thermogravimetric analysis (TGA) was done using a Mettler Toledo TGA/DSC combined instrument and a sample amount of about 10 mg. The heating cycle was from 30 °C to 650 °C at a heating rate of 10 °C/min under nitrogen atmosphere (40 mL min^−1^). Scanning electron microscope (SEM) images (Hitachi S-2700) were taken from both surface and cross-section, obtained by cryogenic fracture, at an accelerating voltage of 15 kV. The samples were placed on a stub and coated with gold. Pore size distribution was calculated by means of FIJI software [35].

### 3.4. Membrane Stability Studies

The stability of the different membranes was investigated by means of mass change. For that, segments of an approximate area of 4 cm^2^ (in the mass range of 0.0225 and 0.0326 g) were cut from different parts of the membrane and were placed in polypropylene vessels containing 50 mL ultrapure water, which were shaken using an orbital mixer for 24 h. Before and after the experiment, membrane segments were carefully weighted. Mass loss is calculated by using Equation (2):

(2) $$Mass\;loss\;\left(\%\right)=\frac{W_{\left(0\right)}-W_{\left(f\right)}}{W_{\left(0\right)}}\times 100$$

where *W*_(0)_ is the initial membrane weight, and *W*_(*f*)_ is the final membrane weight after 24 h immersed in ultrapure water. All experiments were carried out at room temperature of 22 ± 1 °C and were run in triplicate.

### 3.5. Cr(VI) Extraction Experiments

The efficiency of the different membranes to extract Cr(VI) was investigated using membrane segments of approximately 4 cm^2^ size contacted with 25 mL of 10 mg L^−1^ Cr(VI) at pH 4. These conditions ensured an excess of moles of Aliquat 336 over about three times with regards to moles of Cr(VI) in aqueous sample. To ensure the possibility to quantitatively extract the metal, extraction efficiency (Equation (3)) was evaluated by measuring the metal concentration with a sequential inductively-coupled plasma atomic emission spectrometer (ICP-AES) (Liberty RL, Varian, Mulgrave, Vic., Australia):

(3) $$Extraction\;efficiency\;\left(\%\right)=\frac{Cr{\left(VI\right)}_{\left(0\right)}-Cr{\left(VI\right)}_{\left(h\right)}}{Cr{\left(VI\right)}_{\left(0\right)}}\times 100$$

where *Cr*(*VI*)_(0)_ is the initial metal in the aqueous solution and *Cr*(*VI*)_(*h*)_ is the metal concentration in the source solution after a certain time.

All experiments were carried out at room temperature of 22 ± 1 °C and were done by duplicate.

## 4. Conclusions

Satisfactory PIMs incorporating an IL have been prepared by a thermal-compression method, avoiding the use of harmful organic solvents. This novel procedure has led to obtain appropriate membranes based on two different thermoplastic polymers, TPU and PCL, and the IL Aliquat 336, with a fixed content of 70% polymer–30% IL. The characterization of PIMs has revealed that Aliquat 336 was properly included within the polymeric matrix but presenting different interactions depending on the polymer. PCL membranes exhibited a higher stability and a good performance in terms of extraction efficiency, allowing the satisfactory production of high stable membranes based on a biodegradable polymer using a solvent-free preparation method.

## Figures and Tables

**Table 1:** Characteristics of the PIMs prepared with thermo-compression procedure.

Membrane Composition (*w*/*w*)	Characteristics
70% CAP–30% Aliquat 336	Brownish and fragile
70% CAB–30% Aliquat 336	Oily, slightly translucent and fragile
70% PCL–30% Aliquat 336	Non-oily, whitish, translucent and flexible
70% TPU–30% Aliquat 336	Non-oily, whitish, translucent and flexible

**Table 2:** Mass concentration of the characteristic elements of the PIM, presented as the average values percentages (n = 2).

Membrane	Elements	Theoretical (%)	Found (%) (SD)
70% TPU–30% Aliquat336	N	1.4	1.72 (0.04)
	C	68	67.17 (0.02)
	H	10.4	11.1 (0.2)
70% PCL–30% Aliquat336	N	1.03	1.1 (0.2)
	C	64.2	64.4 (0.2)
	H	9.6	9.6 (0.2)

**Table 3:** Chemical structures, melting point and processing temperatures of the different polymers and the IL used for PIM preparation.

**Polymer**	**Chemical Structure**	**Melting Point (°C)**	**Processing Temperature (°C)**
CAP	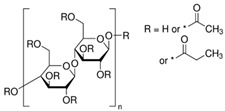	188–210	220
CAB	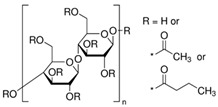	130–160	140
PCL	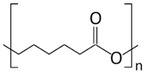	58–60	100
TPU	Hard segment (4%): 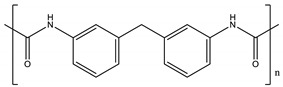 Soft segment (96%): 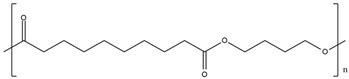	80–84	130
**IL**	**Chemical Structure**	**Melting point (°C)**	**Processing Temperature (°C)**
Aliquat 336	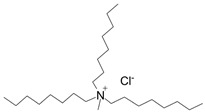	Liquid at room temperature	-
